# Revealing Nafion‐Induced Copper Modifications by Combining In Situ and Ex Situ X‐Ray Absorption Spectroscopy

**DOI:** 10.1002/smll.74606

**Published:** 2026-07-14

**Authors:** Ilargi Napal Azcona, Simone Pollastri, Matteo Bisetto, Manuela Bevilacqua, Federico Salvador, Luca Sbuelz, Michele Zacchigna, Roberto Biagi, Paolo Fornasiero, Silvia Nappini, Elena Magnano

**Affiliations:** ^1^ IOM‐CNR, Istituto Officina dei Materiali A.R.E.A. Science Park Basovizza Trieste Italy; ^2^ Physics Department Università degli Studi di Trieste Trieste Italy; ^3^ Dipartimento di Scienze Fisiche Informatiche e Matematiche, Università degli Studi di Modena e Reggio Emilia Modena Italy; ^4^ Department of Chemical and Pharmaceutical Sciences Università degli studi di Trieste Trieste Italy; ^5^ ICCOM‐CNR Istituto di Chimica dei Composti OrganoMetallici Firenze Italy; ^6^ ICCOM‐CNR Trieste Research Unit at Department of Chemical and Pharmaceutical Sciences Università degli studi di Trieste Trieste Italy; ^7^ Centro H2‐MORE Università degli Studi di Modena e Reggio Emilia Modena Italy; ^8^ Istituto Nanoscienze (NANO‐S3) Consiglio Nazionale delle Ricerche (CNR) Modena Italy

**Keywords:** CO_2_ electrocatalysis, copper, in situ, Nafion, XAS

## Abstract

The electrochemical CO_2_ reduction reaction (CO_2_RR) on a Cu‐based catalyst has garnered significant attention, primarily due to the ability of copper to produce multi‐carbon products. Here, in situ soft X‐ray absorption spectroscopy (XAS) characterization is employed to investigate the dynamics of a copper‐based catalyst during CO_2_RR, probing for the first time the impact of the Nafion commonly applied as a proton‐conductive binder to ensure mechanical integrity and minimize nanoparticles detachment, on the initial oxidation state of the Cu‐catalyst. The results show that Nafion alters the chemical environment of pristine Cu catalyst, leading to the formation of Cu^2+^ species, likely through partial dissolution induced by the acidic nature of the ionomer, followed by coordination of the dissolved copper species with the sulfonic groups of Nafion. These results are corroborated with ex situ Cu K‐edge measurements. The Faradaic efficiency measurements reveal product selectivity differences when Nafion is added by spin‐coating or drop‐casting: while both approaches favor HCOOH as the primary product, spin‐coating enhances CO formation and facilitates ethylene generation. These findings underscore the dual role of Nafion as both a structural binder and an active modifier of first‐stage catalytic behavior, demonstrating the importance of in situ XAS for elucidating the catalyst‐binder interactions during CO_2_RR.

## Graphical abstract

This work elucidates that Nafion alters Cu‐based CO_2_RR catalysts by promoting partial Cu dissolution and stabilizing Cu^2+^ species through coordination with sulfonate (‐SO_3_^−^) groups. Combining in situ Cu L‐edge with ex situ Cu K‐edge XAS measurements, the study highlights how Nafion primarily modifies the nanoparticle surface, while less oxidized Cu fraction remains in the bulk.

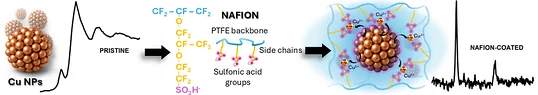

## 1 Introduction

The rising levels of atmospheric CO_2_ over the last decades is one of the primary drivers of climate change, leading to severe environmental consequences. In this context, the development of effective CO_2_ mitigation strategies is of critical importance. In recent years, the electrochemical conversion of CO_2_ to value‐added chemicals and fuels has gained significant attention as a promising approach to achieve a close carbon cycle through the use of renewable energy [1, 2, 3]. CO_2_ is a chemically inert molecule, which is why catalysts are required to activate and convert it into higher‐value products [4]. Among various mono‐metallic catalysts, copper is the only metal capable of producing hydrocarbons, like methane, ethylene, and ethanol, directly from the CO_2_ molecule, distinguishing it from other pure metal catalysts [5]. Therefore, considerable effort has been made to understand the unique catalytic properties of copper and uncovering the mechanisms behind hydrocarbon formation.

Nevertheless, a lack of selectivity in the performance of Cu‐based electrocatalyst during CO_2_RR, still results in a wide range of products, including C_1+_ products (e.g., CO, HCOOH, HCHO, and CH_4_) and C_2+_ hydrocarbons (e.g., C_2_H_4_, C_2_H_5_OH, CH_3_CHO, CH_3_COO^−^) [6]. Several factors, such as physicochemical properties (e.g., particle size, morphology), chemical oxidation state (Cu^0^, Cu^1+^, Cu^2+^), and even the preparation method, have been reported to influence the catalytic activity [7, 8, 9, 10]. Moreover, in aqueous media, CO_2_RR competes with hydrogen evolution reaction (HER), where H_2_ formation often dominates CO_2_ reduction at low overpotentials [11]. As a result, recent research on Cu‐based CO_2_RR electrocatalysts has focused on controlling product distribution by modifying the physicochemical properties of the catalyst.

Understanding and controlling the dynamics of electronic properties at the solid‐liquid interface during electrochemical CO_2_ reduction reactions (CO_2_RR) is essential for catalyst optimization. This has led to a growing demand for in situ and *in operando* analysis in real working conditions. Synchrotron‐based X‐ray absorption spectroscopy (XAS) technique provides important information about reactions that, like CO_2_RR, involve the transfer of one or more electrons from a solid substrate to a solution or vice versa. Soft‐XAS provides valuable insights into catalyst properties, offering high surface sensitivity, element‐specific detection, strong sensitivity to the oxidation state of transition metals, and the ability to probe the chemical state of light elements such as carbon, nitrogen, phosphorus and oxygen. These capabilities enable precise identification of the catalyst chemical state and the reactive intermediates involved during the reaction. All these features make soft‐XAS an invaluable tool for investigating surfaces and solid‐liquid interfaces, offering a unique opportunity to enhance our understanding of the mechanisms governing CO_2_RR on Cu‐based catalysts. On the other hand, high energy XANES can provide complementary information, such as bulk‐sensitive insights into the local atomic structure, coordination environment, and oxidation state of the elements. When combined, soft and hard XAS can provide a comprehensive picture of both surface and bulk properties of the catalyst, enabling a complete understanding of the structure‐activity relationships and contributing to the optimization of selective and efficient catalytic systems.

In our recent work, advanced in situ soft‐XAS has been employed to deepen our understanding of the electronic and chemical properties of Cu‐based catalysts during CO_2_RR [12, 13]. However, it is equally important to consider the role of other components of the electrocatalytic system that can influence the catalyst behavior under operating conditions. One key element is the binder, which is commonly used to provide mechanical/physical stability and adhesion of the catalyst layer to the electrode, whether at the laboratory scale or for industrial‐scale CO_2_RR electrolyzes [14]. Because of the complex nature of binders and the wide range of experimental conditions, conflicting results have been reported in the literature regarding the impact of specific binder properties, such as wettability, which can influence the mass transfer of the key reactants in the CO_2_RR, that is, CO_2_ and H_2_O, thereby affecting the final performance [14, 15, 16, 17, 18, 19, 20]. Despite their essential function, the direct impact of binders on the chemical state of the catalyst and on the reaction mechanism itself remains poorly understood. In this context, a deeper investigation into the chemical interactions between binders and catalytic materials is urgently needed to optimize CO_2_RR systems more rationally.

In the present study, the main goal was to investigate the chemical interaction between the commonly used Nafion binder and the electrodeposited Cu‐catalyst to determine how this interaction can modify the catalyst's initial chemical composition, electronic structure, and consequently its CO_2_RR performance. Nafion is a sulfonated tetrafluoroethylene‐based fluoropolymer composed of a chemically robust polytetrafluoroethylene (PTFE) backbone and perfluorinated side chains terminated with sulfonic acid groups (–SO_3_H), which provide both excellent chemical stability and high proton conductivity. Here, we provide new insights into how Nafion influences the chemical environment of pristine catalytic material, previously scarcely explored in the literature. Ex situ XAS measurements at the Cu L‐ and K‐edges revealed that the Nafion binder modifies the oxidation state of the initial copper catalyst (electrodeposited Cu NPs), leading to Cu^2+^ formation that may arise from partial Cu dissolution induced by the acidic nature of Nafion solution. Complementary in situ soft XAS further demonstrated a gradual re‐deposition process of these Nafion‐associated Cu^2+^ species into metallic Cu under CO_2_RR operating conditions. Additionally, differences in product selectivity were observed depending on whether Nafion was added via spin‐coating or drop‐casting methods onto the Cu NPs. In both cases, HCOOH was the predominant product, with Faradaic efficiency (FE) of approximately 36% at a potential of −1.1 V versus reversible hydrogen electrode (RHE). However, the spin‐coated approach led to enhanced CO formation and enabled the generation of ethylene.

## 2 Results and Discussion

### 2.1 Ex Situ XAS Characterization

Cu NPs were synthesized via electrodeposition and coated with Nafion ionomer (0.75 wt.%) to improve the catalyst stability during CO_2_RR. Two different Nafion coating methods were investigated: drop‐casting and spin‐coating.

**FIGURE 1:**
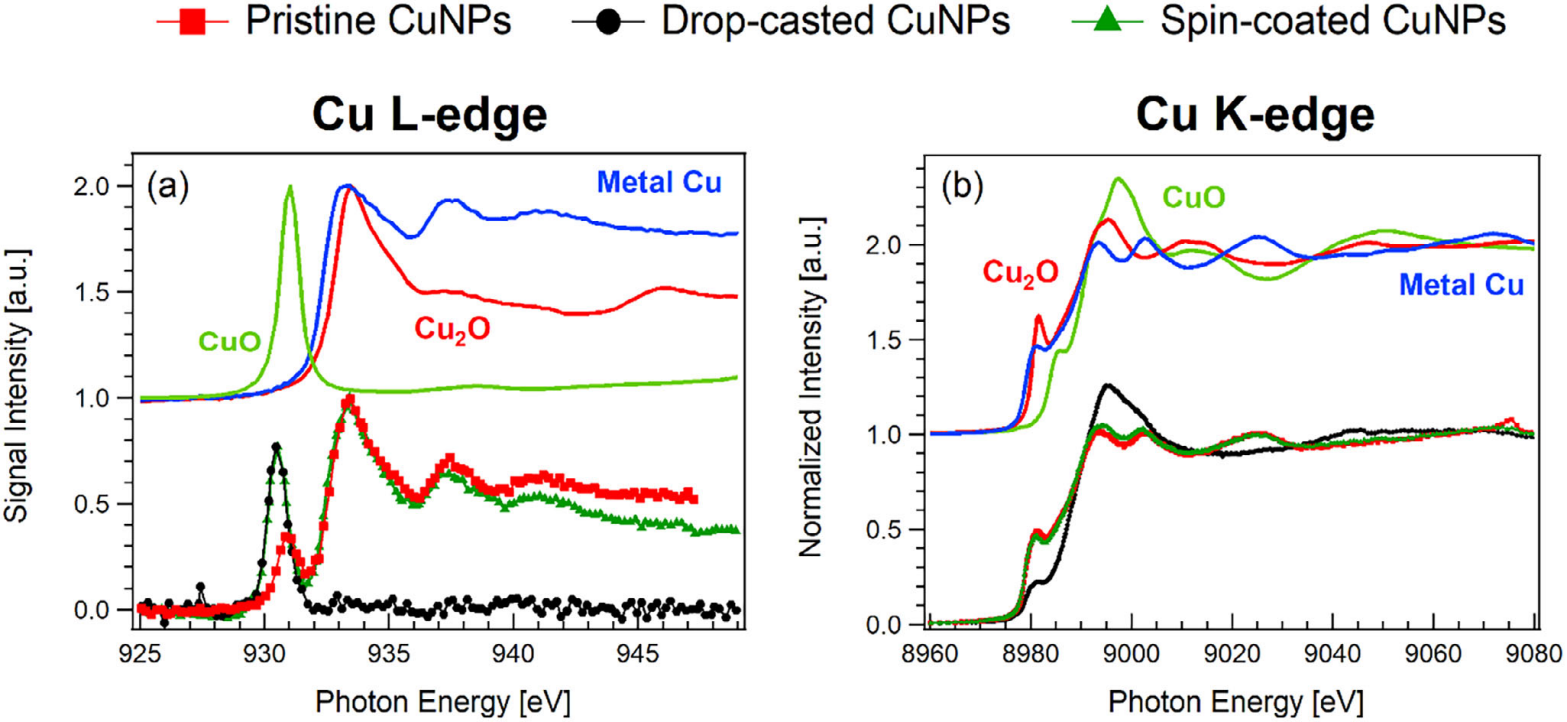
Ex situ XAS measurements: (a) Cu L_3_‐edge and (b) Cu K‐edge of the as‐prepared Cu NPs (red square), drop‐casted (black circle) and spin‐coated (green triangle) CuNPs with 0.75 wt.% Nafion ionomer. Reference spectra of metallic Cu, Cu_2_O, and CuO are shown at the top part for comparison.

Figure 1 presents the ex situ XAS spectra at the Cu L_3_‐edge (Figure 1a) and K‐edge (Figure 1b) for Nafion‐coated (drop‐casted and spin‐coated) and uncoated samples, along with reference spectra of metallic Cu, Cu_2_O, and CuO standards. The L_3_‐edge spectra were acquired in Total Fluorescence Yield (TFY) mode, whereas the K‐edge ones were collected in Partial Fluorescence Yield (PFY) mode for the experimental samples, and in transmission mode for the reference compounds.

The uncoated Cu catalyst (pristine Cu NPs) exhibits a mixed oxidation state. The Cu L_3_‐edge spectrum shows dominant features associated with metallic Cu and Cu_2_O at 937.8 and 933.4 eV respectively, along with a minor contribution of CuO, as indicated by the peak at approximately 931 eV. This picture is confirmed by Cu K‐edge XAS data, where the Linear Combination Fitting (LCF) analysis quantifies the pristine catalyst composition as 81% (± 1) of metallic Cu, 18% (± 2) as Cu^1+^ and 1% (± 0.3) as Cu^2+^ (Figure S2a and Table S1).

A clear change in copper oxidation state upon Nafion coating has been revealed by both L_3_‐ and K‐edge XAS spectra. Specifically, the Cu L_3_‐edge spectrum of the drop‐casted sample shows a single feature at ∼930.45 eV, characteristic of Cu^2+^ oxidation state, shifted by 0.55 eV to lower photon energy compared to the reference CuO signal. This energy downshift suggests a change in the local chemical environment around Cu atoms.

Importantly, all samples were prepared under identical conditions and exposed to air for a comparable duration prior to Nafion deposition for two of them. Therefore, while ambient exposure contributes to a baseline oxidation of ex situ prepared Cu nanoparticles, it cannot account for the strong differences observed among Nafion‐coated samples. Instead, the enhanced Cu^2+^ contribution after Nafion deposition supports that the Nafion coating plays the dominant role in driving the changes in the CuNPs oxidation state. Specifically, the sulfonic acid (–SO_3_H) groups generate a locally acidic environment that promotes partial dissolution of Cu, followed by stabilization of the resulting Cu^2+^ species through coordination with sulfonate (–SO_3_^−^) groups within the polymer network.

This picture is confirmed by K‐edge XAS spectra, where the spectrum of the drop‐casted sample exhibits an increased white line intensity, pointing to the presence of oxidized Cu species, different with respect to that of CuO (different shape and energy position). As shown in Figure S3b, the different reference compounds exhibit characteristic spectral fingerprints. Metallic Cu displays a pronounced low‐energy pre‐edge contribution and an earlier edge onset, while CuSO_4_·5H_2_O, where Cu^2^^+^ is coordinated in a distorted octahedral environment [21], shows an almost absent pre‐edge and a sharp white‐line at higher energy. In contrast, CuO exhibits a distinct shoulder at the rising edge at ∼8985.5 eV, commonly associated with its square‐planar coordination geometry and specific Cu–O bonding interactions [22].

Importantly, the spectrum of the Nafion‐treated drop‐casted Cu NPs does not reproduce the CuO shoulder feature, despite showing Cu^2^^+^‐like characteristics in the edge position and white‐line region. Instead, its spectral profile is more consistent with Cu^2+^ species in a disordered solvated coordination environment, closer to that of CuSO_4_ than to crystalline CuO. This supports our interpretation that part of the copper is dissolved by the acidic Nafion medium and stabilized as Cu^2^^+^ species through coordination with the sulfonic groups within the ionomer matrix, rather than being converted into a CuO phase. Moreover, the weak pre‐edge intensity is also consistent with a more ionic and less covalent Cu–ligand interaction [23], similar to that observed for solvated Cu^2+^ species in CuSO_4_·5H_2_O. Nevertheless, a fraction of metallic Cu is still present, as suggested by the low‐energy feature at about 8980 eV.

The absence of detectable metallic Cu signals in the corresponding Cu L_3_‐edge XAS spectrum indicates that unoxidized copper is present in the core of the NPs, but remains inaccessible to soft X‐rays, because of the intrinsically short attenuation length at 935 eV (∼100 nm, much smaller than the ∼200 nm particle diameter) combined with the additional signal attenuation caused by the Nafion overlayer, limiting the effective probing depth to only the outermost region of the sample. Therefore, the combined hard and soft XAS results suggest that Nafion primarily modified the surface and near‐surface chemical state of the Cu nanoparticles by stabilization of Cu^2+^ species within the sulfonate‐rich ionomer environment, while less oxidized Cu fraction remains in the bulk of the nanoparticles.

The Cu K‐edge XAS LCF analysis of the drop‐casted Nafion sample was performed using reference spectra of metallic Cu, Cu_2_O (Cu^1+^), CuO (Cu^2+^) and CuSO_4_ (Cu^2+^). Notably, CuSO_4_ was specifically included in the fit as a model compound to represent Cu^2+^ species coordinated with the ‐SO_3_H group of Nafion, for the reasons discussed above. In addition, the Cu L‐edge spectrum of drop‐casted CuSO_4_ closely matches that of the Cu^2+^‐Nafion species (Figure S3a), clearly showing that the sample peak aligns with the CuSO_4_ reference. This result also provides strong evidence for the formation of a chemical bond between the polymer and the copper. The LCF results (Figure S2b) show that Nafion‐coated samples exhibit a composition of 24% (± 1) metallic Cu, 5% (± 3) Cu^1+^, and 71% Cu^2+^, distributed as 48% (± 1) CuSO_4_‐like species and 23% (± 2) CuO. The high content of CuSO_4_ ‐like species strongly support the hypothesis that the chemical interaction between Cu NPs and the functional groups of Nafion occurs through the ‐SO_3_H functionalities and plays a significant role in promoting Cu oxidation. Moreover, the Cu K‐edge XAS of the spin‐coated sample exhibits a spectral lineshape more closely resembling that of the uncoated NPs, in agreement with the soft XAS measurements.

The same downward shift of the Cu^2+^ component in the L_3_‐edge spectrum was observed in the spin‐coated sample. However, this sample also exhibits a strong contribution from Cu_2_O and a small amount of metallic Cu. The different surface chemical composition observed between the drop‐casted and spin‐coated Nafion samples are attributed to the dispersity in layer thickness. The thickness of the drop‐cast film was estimated to be ∼1.36 µm (see SI for details), while the spin‐coated layer is expected to be ∼100 nm, consistent with previous reports [19, 24, 25]. It should be noted that both coatings are not perfectly uniform, and local thickness can vary across the sample. Soft X‐rays in the 925–965 eV energy range can penetrate approximately 1 µm of Nafion layer before losing the ∼63% of their intensity [26]. Consequently, X‐rays can fully penetrate spin‐coated Nafion layer and nearly the entire drop‐cast layer. This accounts for the difference observed in the Cu L‐edge spectra between drop‐cast and spin‐coating samples.

The transmission of the outcoming photons is strongly attenuated for the drop‐casted sample, thus, the detected signal predominantly originated from the outermost region of the nanoparticles. In contrast, the thinner spin‐coated Nafion layer causes minimal attenuation, enabling X‐rays to penetrate more, resulting in spectral features similar to those of pristine sample. Importantly, soft XAS data highlight a small contribution of Cu^2+^ species in the spin‐coated samples matching the spectral component observed in the drop‐cast film. This confirms the formation of copper species distinct from CuO, but attributable to surface Cu^2+^ atoms coordinated with Nafion, most likely through the SO_3_H groups, as suggested by the Cu K‐edge analysis. Indeed, a tiny fraction of CuSO_4_ (5% ± 1), with the rest being 72% (± 1) metal, 18% (± 1) Cu_2_O and 5% (± 1) CuO is revealed by LCF results on the K‐edge spectrum of the sample (Figure S2c).

The interaction between Cu^2+^ species and copper atoms was further supported by FTIR and contact angle measurements. Although Nafion binder contains both hydrophilic and hydrophobic groups, it is usually considered a hydrophobic binder [14]. Contact angle measurements (Figure S4) of Nafion‐coated Cu NPs reveal a hydrophobic surface, suggesting that the hydrophilic sulfonic acid (‐SO_3_H) groups may interact with the surface copper atoms, forming Cu^2+^ species, leaving the hydrophobic PTFE chains exposed on the surface. Moreover, FTIR measurements showed that the ν_s_ (SO_3_^−^) band exhibited a slight red shift from 1056.5 to ∼1055.3 cm^−^^1^ together with the appearance of an additional feature at ∼1041 cm^−^^1^, for the Nafion‐coated CuNPs, suggesting a modification of the local sulfonate environment consistent with interactions between Nafion and Cu surface species (Figure S5).

### 2.2 In Situ Soft‐XAS Characterization

The evolution of a Cu electrocatalyst coated with drop‐casted Nafion layer was studied during CO_2_RR by in situ soft‐XAS at the Cu L_3_‐edge collected in TFY mode in CO_2_‐saturated 0.1 M KHCO_3_ electrolyte, using the microfluidic electrochemical cell (MEC‐cell) available on the BACH beamline at Elettra Sincrotrone Trieste (see Figure S1 and SI for details). This in situ approach could enable the direct correlation between the catalytic behavior (i.e., product selectivity) of a copper electrode and its electronic structure modification under similar reaction conditions. In Situ, Cu L_3_‐edge TFY‐XAS spectra of the copper electrode were measured over a potential range from open circuit potential (OCP) to ‐0.9 V vs. RHE.

Three CO_2_ reduction cycles were carried out: (1) the as‐prepared Nafion‐coated Cu NPs, without previous electrochemical voltammetry cycles (CV), avoiding any possible modification of the catalyst; (2) after leaving the system to turn back to OCP, to evaluate the reversibility and stability of the system; and (3) after anodic oxidation of Cu NPs at 1.1 V vs. RHE, to assess the influence of initial Cu oxidation state on CO_2_RR behavior.

**FIGURE 2:**
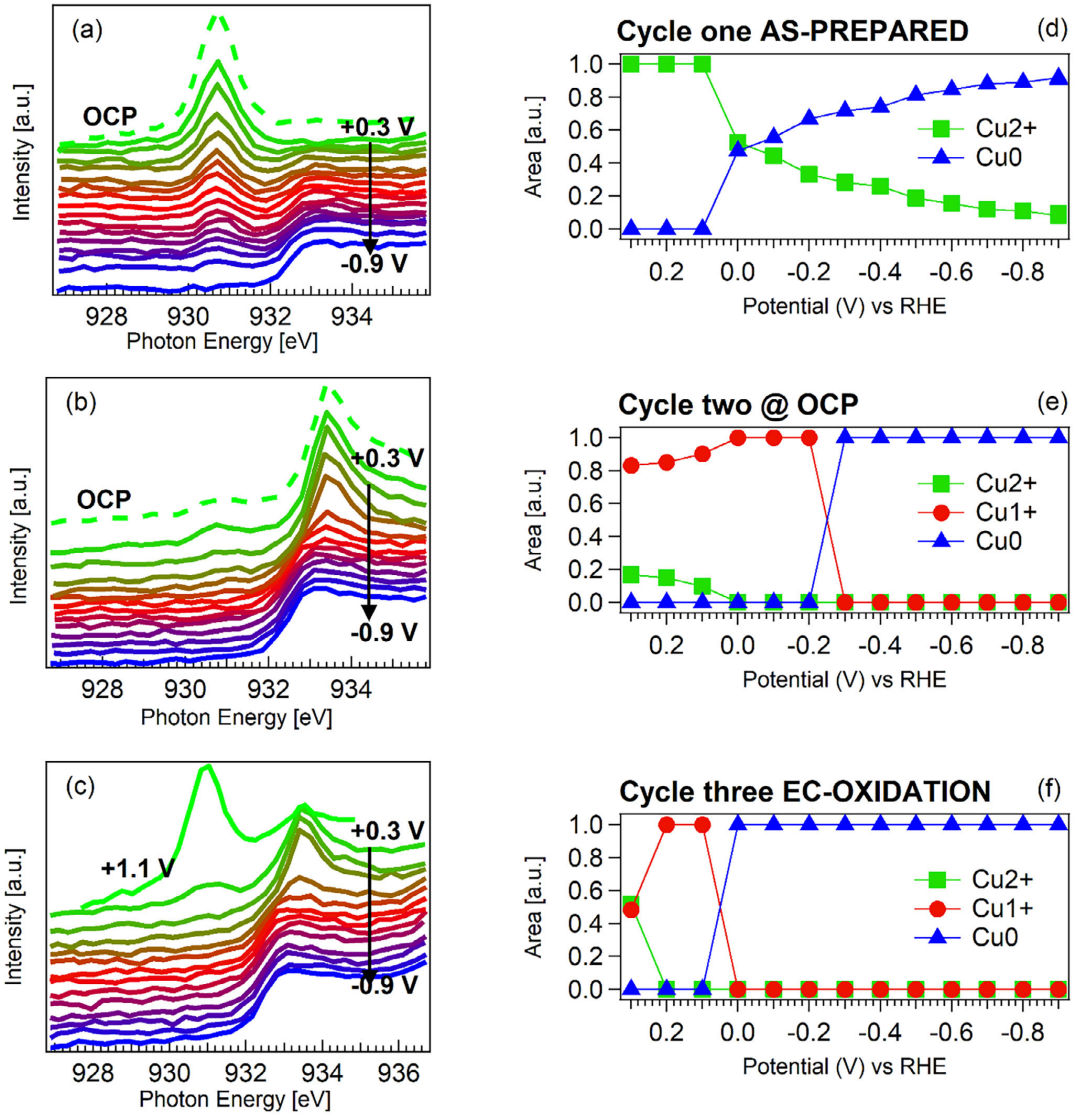
In Situ Cu L_3_‐edge TFY‐XAS measurements for the first (a), second (b) and third (c) CO_2_RR experiments, and the corresponding analysis of the Cu oxidation states (Cu^0^, Cu^1+^ and Cu^2+^) evolution as a function of the applied potential for the first (d), second (e), and third (f) cycles, respectively. Connecting lines have been added between data points for visual clarity.

Figure 2a and Figure 2d present the Cu L_3_‐edge spectra and the corresponding evolution of the areas associated with the different Cu oxidation states as a function of the applied potential during the first reduction cycle. These areas were estimated by an approximate Gaussian deconvolution of the Cu L_3_‐edge spectra (see Figure S9 for more details). In agreement with the ex situ XAS measurements, the copper catalyst under OCP conditions appears primarily in the Nafion‐stabilized Cu^2+^ oxidation state. As the applied potential progressively becomes more negative, the peak related to Cu^2+^ decreases, while the peak related to metallic Cu gradually grows. The evolution of the oxidation state peak areas in Figure 2d confirms that a direct reduction from Cu^2+^ to metallic Cu occurs progressively, with no detectable intermediate formation of Cu^1+^ species. This is consistent with the direct re‐deposition of Cu^2+^ species dissolved in Nafion into their metallic form.

Moreover, even at −0.9 V vs. RHE, a catalytic potential relevant for the CO_2_ reduction, the Cu^2+^ signal persists for several minutes before its reduction to metallic Cu, suggesting a strong electrochemical stability of the Nafion‐Cu^2+^ chemical coordination. This potential range in CO_2_‐saturated KHCO_3_ solution (pH 7–7.5) corresponds to the region where key CO_2_RR products typically form on Cu‐based catalysts, when copper is expected to be in its metallic state Cu(0) [27, 28, 29]. The complete re‐deposition of metallic Cu was achieved only after 10 min of applying the cathodic potential (Figure S6a), when the surface can reasonably become catalytic active.

Once the system returned to OCP conditions, the copper nanoparticles (Cu NPs) mainly exhibit Cu^1+^ oxidation state, with some minor Cu^2+^ contribution, as shown in Figure 2b, suggesting a partial recovering of the pristine Cu oxidation state prior to Nafion coating. This result implies that the first CO_2_RR disrupts the original chemical coordination between Cu and the sulfonic acid groups of Nafion, allowing the re‐deposition of Cu NPs into their metallic form. After this first cathodic sweep, the local environment and pH is expected to change substantially, especially considering the very small electrolyte volume used here (ca. 20 µL). In this reconditioned state, as expected from the Pourbaix diagram [30], the predominance of Cu^1+^ at OCP becomes plausible and is consistent with the expected Cu_2_O stability in a slightly more alkaline local environment at a potential close to 0 V vs SHE.

In the second CO_2_RR cycle, the Cu^1+^ signal reduced to metallic Cu at a well‐defined potential, ca. −0.1 V vs. RHE (Figure 2e), ultimately reaching a fully metallic state at a less negative potential (−0.4 V vs. RHE) compared to the first cycle. Upon returning to OCP conditions, the Cu NPs continue exhibiting a Cu^1+^ electronic structure, indicating that this is the stable oxidation state after the Nafion‐Cu coordination breakage in the first activation cycle.

To evaluate how the initial oxidation state of the catalyst influences the reduction kinetics of copper during CO_2_RR and assess the reversibility of the Nafion‐Cu interaction upon exposure to an oxidative potential, a third cycle was conducted following the prior oxidation of Cu NPs at an anodic potential of 1.1 V vs. RHE. The results show a clear difference between these two species. Figure 2c and Figure 2f illustrate the evolution of Cu electronic structure during the third CO_2_ reduction cycle, while Figure S6b compares the Cu L‐edge spectra of the initial Nafion‐coated Cu sample, the post‐cycle Cu sample at OCP, and the EC oxidized Cu, alongside a CuO reference spectrum. The spectra clearly show that the Cu^2+^ species formed by EC oxidation are shifted to higher photon energy compared to the initial Nafion‐Cu^2+^ species, consistent with CuO‐like / Cu‐OH‐like formation. This reveals two distinct initial states for the CO_2_RR cycles: a Cu^2+–^Nafion bonded state in the first cycle and CuO in the third one. Consistently, the electrochemical behavior of the two Cu^2+^ species differs substantially.

In Situ, XAS in the third cycle confirms that the EC formed copper oxide is more easily reducible than the original Nafion–Cu^2^^+^ system: upon applying a potential of 0.3 V vs. RHE, the Cu^2+^ oxide signal vanishes instantaneously. This suggests that the Cu^2+^ signal observed in the Nafion‐coated sample does not exclusively correspond to a surface oxide, but to Cu^2+^ species trapped within the Nafion layer, which are progressively reduced and redeposited as Cu^0^ during the first cathodic scan.

In contrast to the first cycle, a Cu^1^^+^ intermediate (assigned to Cu_2_O) is clearly observed during reduction, indicating a stepwise reduction pathway. This third cycle closely resembles the second one, indicating that the electronic evolution of Cu under CO_2_RR is influenced not by its initial oxidation state, but by the presence or absence of chemical coordination with Nafion. Once the Nafion–Cu chemical interaction is disrupted during the first CO_2_RR cycle, the catalyst follows a different redox route and strongly stabilizes the Cu^1+^ oxidation state at OCP. Additionally, in the third experiment, the reduction of Cu^1+^ to metallic Cu takes place at a less negative potential (∼0 V vs. RHE) compared to the second experiment. This shift indicates that successive activation cycles can facilitate the formation of catalytically active Cu^1+^ and Cu^0^ species at less negative potentials. As a result, the CO_2_RR can potentially proceed closer to the theoretical conditions, effectively lowering the overpotential. Operating at less negative potentials also suppresses the competing HER, enhancing selectivity toward CO_2_ reduction and improving the overall CO_2_ electrocatalytic efficiency.

It is worth noting that the stability of the sample seems not to be affected by the breakage of the Cu‐Nafion chemical coordination. In fact, the Nafion layer preserves a good adhesion of the Cu catalyst to the electrode substrate, limiting the complete dissolution of the material.

### 2.3 Catalytic Performance of Nafion‐Coated Cu Nanoparticles

**FIGURE 3:**
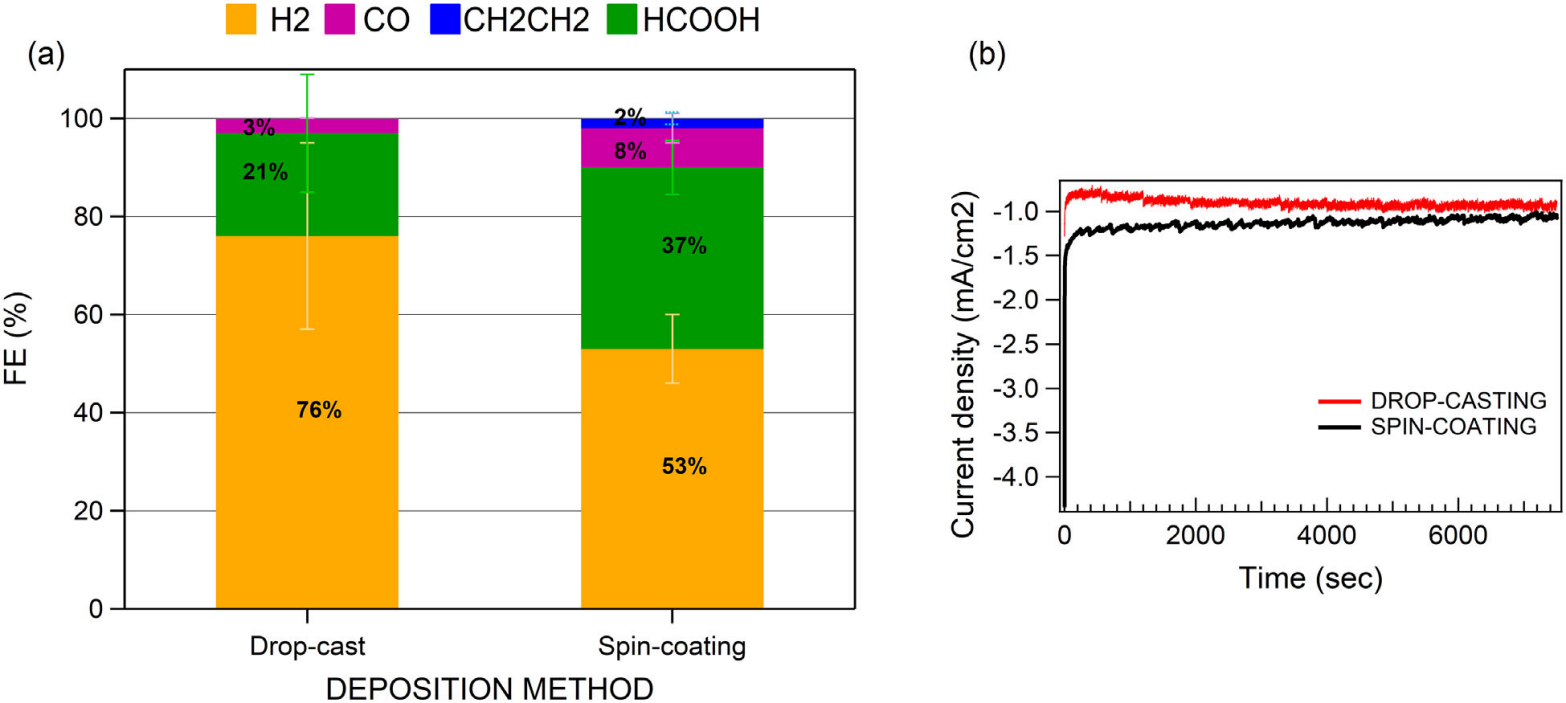
(a) Resulting FE in the H‐cell at −1.1 V vs. RHE, and (b) corresponding chronoamperometry over the CO_2_RR experiment for Nafion 0.75 wt.% drop‐casted and spin‐coated Cu NPs. Error bars represent the standard deviation of measurements based on three independent samples for spin‐coated samples and two independent samples for drop‐casted samples.

The catalytic performance of the Nafion‐coated Cu NPs catalyst was performed with an H‐cell setup connected to an online gas chromatograph (GS), after applying a sufficient number of activation cycles to ensure that the initial difference in oxidation state no longer plays a significant role. Under these conditions, Cu NPs are expected to be predominantly in the Cu^1+^ state at OCP, as suggested by the in situ XAS measurement. Both Nafion coating approaches (spin‐coating and drop‐casting) were used for the sample preparation. Figure 3a and Figure 3b show the overall Faradic efficiency (FE) and current density developed over 120 min of chronoamperometry (CA) at −1.1 vs. RHE. In both cases, a similar and stable current density of about −1 mA cm^−2^ was measured for the whole process. The GS analysis revealed that both samples predominantly produce H_2_, suggesting a high concentration of the Cu‐H species located at the catalyst surface. However, this product is lower for the spin‐coated sample, which exhibits a significant increase in CO production (FE ∼11% vs. 3%). Moreover, the liquid products analysis indicates good selectivity toward HCOOH (formic acid), with slightly higher FE values for the spin‐coated sample (∼38%). Contact angle measurements (Figure S4) confirmed that both Nafion coating methods resulted in similarly hydrophobic surfaces, consistent with literature that correlates increased HCOOH production with enhanced desorption of liquid products due to surface hydrophobicity [31, 32].

Usually, a higher FE_CO_ is associated with ethylene production, as the formation of *CO species can enhance the C‐C coupling, promoting the generation of C_2+_ products [33], as observed in the spin‐coated sample. However, the hydrophobic nature of the sample surface, due to the Nafion coating, can hinder proton transport, promoting the desorption of the *CO intermediates and favoring the release of CO gas rather than further hydrogenation toward C_2+_ products [18]. This explains the low FE values obtained for ethylene (∼3%) compared to CO (∼11%).

Chou and co‐workers reported a correlation between the presence of Cu^0^ and Cu^1+^ species and the enhanced C_2+_ production [34]. In this work, the in situ XAS experiments demonstrated the formation of stable Cu^1+^ species after activation cycles, which were also performed prior to the CA measurements to determine the FEs. As discussed above, this process led to the disruption of Nafion‐Cu chemical interaction. Furthermore, the conversion to fully metallic copper was observed at potential before the onset of catalytic activity, suggesting the stabilization of active Cu oxidation states for the C_2+_ production.

However, the observed differences in product selectivity between the spin‐coated and drop‐cast samples cannot be explained by oxidation state alone. The thickness and uniformity of the Nafion layer can also play a crucial role, as reported in literature [19, 35]. Spin‐coating allowed for more precise control of layer thickness, whereas drop‐casting resulted in a thicker, non‐uniform coating, which likely limits the CO_2_ mass transport to the electrode surface due to the lower permeability [32, 36].

Repeated FE experiments (Figure S7) further demonstrated that the drop‐cast method exhibited significant variability in product selectivity, reflecting a not uniform Nafion coverage and different thickness profile across the sample. In contrast, spin‐coated samples yielded consistent and more reproducible results, confirming the method's reliability in achieving uniform and thinner coatings.

The coating process likely alters the microstructure of the catalyst covering the electrode, thereby affecting its selectivity. Scanning electron microscopy (SEM) images of drop‐cast samples (Figure S8b) show a denser Nafion coverage compared to the spin‐coated ones (Figure S8c), resulting in nanoparticle agglomeration and formation of large clusters. The presence of the thick polymer layer hinders deeper CO_2_ diffusion, which can favor the HER process, a competing reaction that does not require CO_2_. Additionally, the nanoparticles agglomeration reduces the catalytic surface area available for CO_2_RR, decreasing the catalyst performance toward CO and HCOOH production.

In contrast, the spin‐coated sample maintains the initial Cu NPs morphology with a uniform small‐size particle distribution across the electrode surface, resulting in a larger available catalytic surface area. This suggests that the thinner layer of Nafion formed by spin‐coating promotes more favorable micro‐environmental conditions for *CO adsorption, a key intermediate in C_2+_ product formation. Furthermore, the thinner Nafion layer facilitates the diffusion of products like HCOOH and CO, thereby enhancing the final FE values.

## 3 Conclusion

Nafion coating significantly alters the initial chemical state and catalytic behavior of copper‐based catalysts during CO_2_RR. In Situ XAS measurements demonstrate the formation of a strong chemical interaction between Cu and Nafion, attributed to a dissolution process induced by the acidic nature of the Nafion, that stabilized Cu^2+^ species into the Nafion matrix through interaction with sulfonic acid groups. During the first cathodic sweep under CO_2_RR conditions, copper is re‐deposited in its metallic form, disrupting the initial chemical interaction between Nafion and copper atoms. These Cu NPs evolve over EC reduction cycling, leading to the formation of stable Cu^1+^ species (likely Cu_2_O) at OCP.

Importantly, the different structural properties of the Nafion film, determined by the applied deposition technique (drop‐cast or spin‐coating), emerge as a key factor of CO_2_RR selectivity. While the hydrophobic nature of Nafion‐coated Cu surfaces consistently promotes HCOOH formation regardless of the deposition method, only spin‐coated catalysts demonstrate enhanced CO and ethylene production. This improved performance is attributed to the thinner Nafion layer achieved via spin‐coating, which facilitates CO_2_ mass transport to the electrocatalytic interface and maintains a high active surface area by preserving the initial nanoparticle morphology. In contrast, drop‐cast films result in a thicker, non‐uniform coating that hinders CO_2_ diffusion, and promotes the competing HER, causing nanoparticle agglomeration and loss of catalytic surface area. These findings highlight the critical role of both the chemical interaction between Nafion and copper, and the physical characteristics of the Nafion layer in determining the selectivity and efficiency of CO_2_ electroreduction.

## 4 Experimental Section/Methods

### 4.1 Synthesis and Preparation of Copper Electrode

The catalytic material was electrodeposited onto an Au/Ti‐coated SiN_x_ membrane (100 nm thick 1×1 mm^2^ window, frame thickness 200 µm; Ted Pella, Prod # 21905, which acts as working electrode (WE) window in the MEC cell for soft‐XAS experiments (see the cell schematic in Figure S1 and SI for more details). Prior to the sample deposition, a thin film of Ti (3 nm) was deposited by physical vapor deposition (PVD), followed by deposition of 15 nm of Au via PVD, obtaining a conductive WE support. Cu NPs were deposited onto this Au/Ti‐coated SiN_x_ membrane by electrodeposition process from an aqueous solution of 10 mM CuSO_4_ ·5H_2_O and 10 mM Na_2_SO_4_/H_2_SO_4_ (Sigma Aldrich) (pH 2). The electrodeposition was carried out in galvanostatic mode, using a constant current density of –1.3 mA/cm^2^ for 40 s. After the electrodeposition, the sample was rinsed with Milli‐Q water to remove any possible CuSO_4_ contamination. This process yields the deposition of Cu NPs with an average size of 150 nm.

To ensure consistency between the in situ XAS experiments and the electrochemical CO_2_RR measurements, the same surface termination of the supporting electrode substrates was used in both setups. Therefore, Au/Ti coated Si wafers were employed as electrode substrates for the study of the catalytic performance during CO_2_RR in the H‐cell setup. For these samples, a deposition time of 200 s was applied to achieve the desired surface coverage and mass loading of ≈ 86 µg cm^−2^. All the samples were prepared under identical conditions and exposed to ambient air for comparable duration prior to Nafion deposition.

The commonly employed Nafion ionomer (Sigma‐Aldrich) was used as a binder to prevent the catalyst detachment from the electrode surface during electrochemical measurements. A Nafion solution of 0.75 wt.% was prepared by diluting the commercial 5 wt.% hydro‐alcoholic Nafion solution with an isopropanol/water (2:1) mixture. Two coating methods were employed for Nafion deposition on the electrodeposited Cu NPs and deeply studied: (1) drop‐casting and (2) spin‐coating. For drop‐casting, a 40 µL aliquot of the Nafion solution was spread on the electrodeposited Cu NPs and dried at room temperature overnight. For the spin‐coating, 40 µL of the solution was deposited onto the Cu NPs, followed by spinning at 750 rpm for 30 s. A final curing step was performed at 60°C for 2 min to ensure proper adhesion and solvent evaporation.

### 4.2 Ex Situ and In Situ XAS Characterization

Soft‐XAS measurements were performed at the BACH beamline [37] of IOM‐CNR at the Elettra Sincrotrone Trieste (Italy), while hard‐XAS was conducted at the SAMBA beamline [38] of the Soleil Synchrotron (France).

Figure S1 shows the scheme of the microfluidic electrochemical (MEC) cell and the set‐up for in situ soft‐XAS experiments implemented at BACH dedicated to spectro‐electrochemical studies [12, 13, 39]. In Situ, the Cu L‐edge XAS was recorded in TFY mode using a XUV‐100 absolute Si photodiode detector (IRD AXUV100), positioned at an angle of 30° relative to the incident beam and placed 1–2 cm away from the cell to guarantee maximum signal intensity. The spectra were collected during electrochemical CO_2_RR using the MEC‐cell filled with a CO_2_‐saturated KHCO_3_ 0.1 M solution. The Cu L‐edge was probed as a function of applied potential over a potential range from OCP to −0.9 V vs. RHE, providing insights into the unoccupied electronic states of Cu under working conditions. Each potential was applied for 65 s, corresponding to the time required for the acquisition of the Cu L_3_‐edge region spectrum. The electrochemical measurements were conducted using a PamlSens4 potentiostat, capable of operating within a potential range of −5 V to 5 V and a current range of 100 pA to 10 mA.

Ex situ Cu L‐edge XAS was acquired in TFY mode using a multichannel plate (MCP) detector (Hamamatsu, F4655‐13) with an energy resolution better than 0.25 eV. Energy calibration was performed via X‐ray photoemission spectroscopy (XPS) by measuring the kinetic energy of the Au 4f core level from a clean Au plate. Cu L‐edge spectra were processed using Igor Pro 6.37. The reference Cu L‐edge spectra were normalized to the maximum intensity of the main peak. For the ex situ sample spectra (Pristine, Drop‐casted, and Spin‐coated CuNPs), the signal intensity was adjusted for visualization purposes. The in situ spectra were not normalized and are presented as measured. Background subtraction was applied only when required, using a linear baseline correction.

Cu K‐edge XAS was collected in PFY using a 35‐pixel HPGe fluorescence detector; energy calibration was accomplished by simultaneously collecting a reference spectrum of metal Cu foil placed between I1 and I2 ionization chambers, with the position of the first inflection point taken at 8979.0 eV. Spectra from pelletized Cu‐bearing reference compounds were instead collected in transmission mode in the frame of other experiments conducted at the XAFS beamline of Elettra synchrotron [40], using ionization chambers as detectors and a fixed exit double crystal Si (111) monochromator. Cu K‐edge spectra were processed within the XANES region. The XANES spectra of both samples and reference compounds were normalized to an edge step of 1 using pre‐edge linear and post‐edge spline functions implemented in Athena software.

### 4.3 Electrochemical CO_2_RR

The electrochemical CO_2_RR experiments were conducted in a standard electrochemical H‐cell, coupled with an online GS (Agilent Technologies, 7890A), which operates in a three‐electrode configuration. The details of the H‐cell are reported elsewhere [41]. Briefly, the cell consists of a 5×5×5 cm^3^ polycarbonate cube, featuring 1 mm thick platinum wire as a counter electrode (CE), saturated Ag/AgCl as reference electrode (RE) and Nafion‐coated Cu NPs electrodeposited on Au/Ti‐coated silicon wafer as WE. The WE and the CE are placed in separated reaction chambers, divided by a commercial Nafion ion‐exchange membrane. The RE is situated in the same chambers as the WE, to facilitate precise voltage control on the electrode of interest.

During the catalytic experiment, CO_2_ flowed through both compartments of the cell, and the gas‐phase products were collected from the headspace and analyzed using online GC. An offline ion chromatography (IC) analyzer (883 Basic IC Plus, Metrohm) was employed to analyze the liquid products by sampling an aliquot of the electrolyte solution at the end of the catalytic process. The electrochemical tests were conducted with 15 mL of KHCO3 0.1 M (Sigma‐Aldrich ACS reagent, 99.7%) as electrolyte with a CO_2_ flow rate of 10 mL/min for each compartment of the cell. The potential was controlled with an Autolab PGSTAT302N potentiostat.

FE was calculated based on the concentration of the different products over time, using the following equation to determine product formation in both the solution and gas phases:

$$FE=\frac{n_{e}\times n\times F}{Q}$$

Where, *n_e_* is the number of electrons involved in the reaction, *n* is the moles of products, *F* is the Faraday's constant (96485 C mol‐1) and *Q* is the change obtained from the integration of the CA as an absolute value.

### 4.4 FT‐IR Measurements

FT‐IR data have been acquired at the CNR‐IOM Sissi‐Materials offline Laboratory c/o Elettra Synchrotron by using a Bruker 70v spectrometer attached to a Hyperion 2000 microscope. The spectra of pristine Nafion (0.75 wt%) drop‐cast on an AuSi wafer, and the Nafion‐coated CuNPs prepared by spin‐coating and drop‐casting were acquired in reflectance mode, using a bare AuSi electrode as background reference. The resulting spectra were subsequently converted to absorbance units according to $A=-\log_{10}\left(R\right)$, where *R* is the measured reflectance, enabling direct comparison with spectra commonly reported in the literature. We used an aperture of 120 µm after ensuring the homogeneity of the samples and verifying the consistency of the results on different spots on the surface.

## Conflicts of Interest

The authors declare no conflicts of interest.

## Supporting information




**Supporting File**: smll74606‐sup‐0001‐SuppMat.docx.

## Data Availability

The data that support the findings of this study are available from the corresponding author upon reasonable request.
