# Supplementary material for: Revealing Nafion‐Induced Copper Modifications by Combining In Situ and Ex Situ X‐Ray Absorption Spectroscopy
Source: Small. 2026 Jul 14;22(51):e74606. doi: 10.1002/smll.74606 (PMC13568882; doi:10.1002/smll.74606)
Supplement: Supplementary file 1 [file SMLL-22-e74606-s001.docx]

Supporting Information

Revealing Nafion-Induced Copper Modifications by Combining *In Situ* and *Ex situ*

X-ray Absorption Spectroscopy

*Ilargi Napal Azcona, Simone Pollastri, Matteo Bisetto, Manuela Bevilacqua, Federico Salvador, Luca Sbuelz, Michele Zacchigna, Roberto Biagi, Paolo Fornasiero, Silvia Nappini^*^ and Elena Magnano^*^*

I. Napal Azcona, F. Salvador, L. Sbuelz, M. Zacchigna, S. Nappini, E. Magnano

IOM-CNR, Istituto Officina dei Materiali, AREA Science Park Basovizza, 34149 Trieste, Italy

I. Napal Azcona

Physics Department, Università degli Studi di Trieste, 34127 Trieste, Italy

S. Pollastri, R. Biagi

Dipartimento di Scienze Fisiche, Informatiche e Matematiche, Università degli Studi di Modena e Reggio Emilia, 41125 Modena, Italy

R. Biagi

Istituto Nanoscienze (NANO-S3), Consiglio Nazionale delle Ricerche (CNR), Modena 41125, Italy

and

Centro H2-MORE, Università degli Studi di Modena e Reggio Emilia, Modena 41125, Italy

M. Bisetto, P. Fornasiero

Department of Chemical and Pharmaceutical Sciences, Università degli studi di Trieste, 34127 Trieste, Italy

M. Bevilacqua

ICCOM-CNR Istituto di Chimica dei Composti OrganoMetallici, 50019 Firenze, Italy.

M. Bevilacqua, P. Fornassiero

ICCOM-CNR Trieste Research Unit at Department of Chemical and Pharmaceutical Sciences, Università degli studi di Trieste, 34127 Trieste, Italy

E-mail corresponding authors: [nappini@iom.cnr.it](mailto:nappini@iom.cnr.it); [magnano@iom.cnr.it](mailto:magnano@iom.cnr.it)

***Microfluidic electrochemical (MEC) cell for in situ soft-XAS***

The electrochemical set-up used for the *in situ* XAS measurements was developed at BACH beamline to enhanced the original design,[1] with improved performance tailored to studying catalytic materials during electrochemical reactions. The new microfluidic electrochemical (MEC)-cell is a three-electrode system comprising a miniaturized Ag/AgCl reference electrode (RE), a Pt counter electrode (CE), and a working electrode (WE), where the Cu-based catalyst is electrodeposited. The WE consist of a 1 x 1 cm^2^ Au/Ti-coated SiNx membrane frame with 1 x 1 mm^2^, 100 nm thick window that is semi-transparent to X-rays and separates the electrolyte from the ultra-high vacuum (UHV) chamber (**Figure S1**). A Viton O-ring seals the cell yielding an effective WE area of 0.3 cm^2^. All potentials were recorded against the Ag/AgCl (sat.) reference electrode and then converted to reversible hydrogen electrode (RHE) values using the Nernst equation: *E* (vs. RHE) = *E* (vs. Ag/AgCl) + 0.197 V + 0.0591 V × pH.

**Figure S1.** (a) Pictures of the MEC-cell showing the Pt CE, the inlet/outlet cavities for the electrolyte flow, and the Au/Ti-coated SiN_x_ membrane that serves as WE. (b) Schematic drawing of the MEC-cell and the measurements setup for *in situ* soft XAS experiments implemented at the BACH beamline. Images reproduced from: Napal Azcona, I. *X-Ray Spectroscopies for Hydrogen Based Economy* (Doctoral Dissertation).[2]

***Linear Combination Fitting of XAS spectra***

**Figure S2**. Linear combination fitting (LCF) of the XAS spectra of pristine copper sample (a) the Nafion drop-casted sample (b), and the spin-coated sample (c), at the Cu K-edge, using a combination of various Cu-bearing reference compounds. Top graphs displaying a magnified view of the residuals are included to facilitate visual inspection of the fit quality. Hard XAS enables direct probing of the sample composition even through the ~1 µm thick Nafion layer on the drop-cast sample, allowing the identification of all the relevant components (Cu₂O, CuO, metallic Cu and CuSO₄), and demonstrating the coordination of a fraction of Cu²⁺ species to the -SO₃H groups of Nafion.

**Table S1. *Summary of Linear Combination fitting data***

| **SAMPLE** | **LCF coefficients (%)** | | | | ***R*-factor** |
| --- | --- | --- | --- | --- | --- |
|  | **Cu metal** | **Cu_2_O** | **CuO** | **CuSO_4_** |  |
| **Pristine CuNPs (h-XAS)** | 81.2 ± 0.5 | 17.8 ± 1.6 | 1.0 ± 0.3 | - | *0.00038* |
| **Drop-casted CuNPs (h-XAS)** | 24.0 ± 1.2 | 5.1 ± 2.7 | 22.9 ± 1.5 | 48.0 ± 1.2 | *0.00227* |
| **Spin-coated CuNPs (h-XAS)** | 72 ± 1 | 18.3 ± 0.6 | 5.2 ± 0.6 | 5.0 ± 0.5 | *0.00031* |

***Thickness estimation of drop-casted Nafion film***

5 wt.% Nafion solution was diluted in isopropanol-H_2_O mixture (2:1) to obtain a final solution of 0.75 wt.% Nafion. Given the low Nafion concentration, its contribution to the solution density is negligible, so the final solution density can be approximated as that of the solvent mixture (*ρ_final_ ≈ ρ_solvents_*).

Assuming ρ*_IPA_* = 0.785 g mL^-1^ and ρ_H2O_ = 1 g mL^-1^ in a 2:1 volume ratio, the final density is estimated as:

$\rho final\approx\frac{\left( 2\times0.785 \right)+1}{3} \approx0.86 g {mL}^{-1}$.

A volume of 40 µL of the 0.75 wt.% Nafion solution was drop-casted on a 1cm^2^ area, corresponding to a total solution mass of

$0.04 mL\times0.86 g {mL}^{-1}=0.034 g$.

Considering the Nafion mass fraction of 0.0075, the mass of Nafion deposited was

$0.034 g \times0.0075=0.00026g$.

The density of dried Nafion has been estimated in a range between 1.6 – 2.1 g mL^-1^ in literature;[4] here, an intermediate values of 1.9 g·mL^-1^ was selected. The corresponding volume of dried Nafion was

$$\frac{0.00026 g}{1.9 g {cm}^{-3}}=0.000136 {cm}^{3}.$$

Finally, the Nafion film thickness was estimated as:

$$Nafion thickness=\frac{Nafion volume}{total area}= \frac{0.000136 {cm}^{3}}{1 {cm}^{2}}=1.36 \mu m$$

The Nafion film thickness was estimated under the assumption that all the solvent evaporates and the entire Nafion content remains on the coated area.

***Ex situ Cu L-edge and K-edge XAS measurements***

**Figure S3.** (a) Cu L_3,2_-edge and (b) Cu K-edge spectra of drop-casted 10mM CuSO_4_·5H_2_O electrolyte (turquoise square), and Nafion drop-casted Cu NPs (black circle). Metallic Cu and CuO reference spectra are shown in continuous blue and green line, respectively.

***Contact angle measurements***

**Figure S4**. Contact angle measurements of a water droplet on (a) pristine Cu NPs, (b) Nafion 0.75 wt.% drop-casted Cu NPs, and (c) Nafion 0.75 wt.% spin-coated Cu NPs. The results demonstrate that Nafion drop-casting alters the hydrophobicity of pristine copper electrode. In the absence of Nafion deposition, the catalyst surface exhibits a high hydrophobicity with a contact angle of 130°. Moreover, slightly more hydrophilic surface was measured for the spin-coated sample. However, all the surfaces continue to exhibit a certain degree of hydrophobicity, indicating that the -SO_3_H groups may interact with the Cu NPs, leaving the hydrophobic PTFE chains exposed on the surface.

***FT-IR measurements***

As shown in **Figure S5**, all samples exhibit the characteristics vibrational modes of Nafion molecule in agreement with the literature.[4] For all the samples, the peaks assigned to symmetric and asymmetric C-O-C stretching appear at 970 and 981.7 cm^-1^, respectively, while the asymmetric and symmetric stretching modes of CF_2_ chain are observed at approximately 1150 and 1210 cm^-1^, consistent with the values reported in literature.[5]

**Figure S5.** (a) FTIR spectra comparing pristine Nafion (0.75 wt%) drop-cast on AuSi (pink) with Nafion-coated CuNPs prepared by spin coating (green) and drop casting (black). (b) Magnified view of the ν_s_(SO₃⁻) region highlighting changes in the symmetric sulfonate stretching vibration. Spectra were collected at different areas for the Nafion-coated CuNPs, distinguished as dashed and solid lines.

The band located at ~1055-1056 cm⁻¹ has been reported to be highly sensitive to local environment of the SO₃⁻ groups, including ion exchange processes and changes in its polarization state.[6] Therefore, the observed red shift from 1056.5 to ~1055.3 cm⁻¹, in Nafion-coated CuNPs, can likewise be rationalized in terms of electronic polarization effects arising from Cu–sulfonate interactions, which perturb the vibrational modes even of non-coordinated SO₃⁻ groups in their vicinity. [7]

Moreover, in Nafion-coated CuNP samples, an additional contribution appears at 1040–1042 cm⁻¹, which we attribute to SO₃⁻ groups involved in coordination interactions with Cu species at the interface.[8–10] This assignment is consistent with previous reports describing the emergence of a ~1041 cm⁻¹ band in Nafion systems upon interaction of sulfonate groups with ionic species, where the peak was attributed to perturbations of the C–S–O vibrational modes induced by ionic coordination.[11]

The lower intensity of the SO₃⁻ coordinated contribution compared to the main 1056.5 cm⁻¹ band indicates that only a fraction of the sulfonate groups is directly interacting with Cu, while the majority remain unbound, consistent with an interfacial interaction limited to Cu–Nafion contact regions.

Importantly, differences in both peak shape and spectral intensity were observed between different probed regions of the Nafion-coated CuNPs. In the spin-coated samples, these variations were less pronounced than in the drop-casted ones, suggesting a more homogeneous distribution of Nafion in the former case.

***In situ Cu L-edge XAS measurements***

**Figure S6**. (a) *In situ* Cu-L_3_ edge spectra recorded during the 1^st^ CO_2_RR cycle of Cu-Nafion species under catalytic potentials (-0.9 V vs. RHE) and (b) Cu L_3_-edge illustrating various Cu^2+^ species during the CO_2_RR experiments under different conditions: Cu²⁺–Nafion in Nafion coated sample (black), partial oxidation of Cu₂O to CuO under applied anodic potential (yellow), and Cu₂O at open-circuit potential (red) after catalysis. For comparison, the reference spectrum of CuO is shown with green dotted line.

***Reproducibility tests of FE measurements***

**Figure S7**. Resulting FE measurements in the H-cell at -1.1 V vs. RHE for drop-casted and spin-coated samples.

*S****canning electron microscopy characterization of Nafion-Cu NPs***

**Figure S8**. SEM images of Cu NPs electrodeposited on Au/Ti Si wafer: (a) as-prepared, (b) after Nafion coating via drop-casting, and (c) after Nafion coating via spin-coating.

***Gaussian Deconvolution of in situ Cu L-edge XAS Spectra***

**Figure S9**. To obtain a semi-quantitative estimate of the evolution of the Cu species, Gaussian-based spectral decomposition was applied to each XAS spectra obtained during the CO₂RR experiments, with the KolXPD software. This approach is not intended to provide a rigorous physical description of the spectra, but rather to serve as a practical and internally consistent method for tracking relative spectral changes associated with the evolution of Cu oxidation states. The Gaussian components assigned to each copper chemical state were defined according to the photon-energy positions observed in the corresponding reference spectra. While keeping the peak positions of the metallic Cu and the different oxides fixed, the amplitudes were allowed to vary to reproduce the overall spectral envelope of the *in situ* Cu L_3_-edge XAS.

The integrated area associated with the metallic Cu and with each oxidation state was then calculated for every spectrum and normalized to the total area of that spectrum (i.e., the sum of the areas of all Gaussian components). Prior to fitting, all spectra were normalized to their maximum intensity.

***References***

1.	Nappini, S., D’Amario, L., Favaro, M., Dal Zilio, S., Salvador, F., Betz-Güttner, E., Fondacaro, A., Píš, I., Romanzin, L., Gambitta, A., Bondino, F., Lazzarino, M., and Magnano, E. (2021) Soft x-ray spectroscopies in liquids and at solid–liquid interface at BACH beamline at Elettra. *Review of Scientific Instruments*, **92** (1), 015115.

2.	Napal Azcona, I. (2025) X-Ray Spectroscopies for Hydrogen Based Economy. <https://hdl.handle.net/11368/3108003>.

3.	Oberbroeckling, K.J., Dunwoody, D.C., Minteer, S.D., and Leddy, J. (2002) Density of Nafion Exchanged with Transition Metal Complexes and Tetramethyl Ammonium, Ferrous, and Hydrogen Ions: Commercial and Recast Films. *Anal. Chem.*, **74** (18), 4794–4799.

4.	Wei, Y., Qian, T., Liu, J., Guo, X., Gong, Q., Liu, Z., Tian, B., and Qiao, J. (2019) Novel composite Nafion membranes modified with copper phthalocyanine tetrasulfonic acid tetrasodium salt for fuel cell application. *Journal of Materiomics*, **5** (2), 252–257.

5.	Danilczuk, M., Lin, L., Schlick, S., Hamrock, S.J., and Schaberg, M.S. (2011) Understanding the fingerprint region in the infra-red spectra of perfluorinated ionomer membranes and corresponding model compounds: Experiments and theoretical calculations. *Journal of Power Sources*, **196** (20), 8216–8224.

6.	Webber, M., Dimakis, N., Kumari, D., Fuccillo, M., and Smotkin, E.S. (2010) Mechanically Coupled Internal Coordinates of Ionomer Vibrational Modes. *Macromolecules*, **43** (13), 5500–5502.

7.	Karelin, A.I., Kayumov, R.R., Sanginov, E.A., and Dobrovolsky, Yu.A. (2017) FTIR spectroscopic study of the complex formation between H + and DMSO in Nafion. *Spectrochimica Acta Part A: Molecular and Biomolecular Spectroscopy*, **178**, 94–104.

8.	Isa, K.B.Md., Osman, Z., Arof, A.K., Othman, L., Zainol, N.H., Samin, S.M., Chong, W.G., and Kamarulzaman, N. (2014) Lithium ion conduction and ion–polymer interaction in PVdF-HFP based gel polymer electrolytes. *Solid State Ionics*, **268**, 288–293.

9.	Jeong, S.-K., Jo, Y.-K., and Jo, N.-J. (2006) Decoupled ion conduction mechanism of poly(vinyl alcohol) based Mg-conducting solid polymer electrolyte. *Electrochimica Acta*, **52** (4), 1549–1555.

10.	Hambali, D., Zainuddin, Z., and Osman, Z. (2017) Characteristics of novel plastic crystal gel polymer electrolytes based on PVdC-co-AN. *Ionics*, **23** (2), 285–294.

11.	Jadhav, P.S., and Joshi, G.M. (2024) Influence of hybrid filler on charge conduction and storage performance of polyvinyl chloride/nitrocellulose blend for hybrid electrolyte application. *ChemistrySelect*, **9** (11), e202304421.
